# Development of Inert, Polymer-Bonded Simulants for Explosives Detection Systems Based on Transmission X-ray

**DOI:** 10.3390/molecules24234330

**Published:** 2019-11-27

**Authors:** Mitja Vahčič, David Anderson, Miguel Ruiz Osés, Grzegorz Rarata, Gabriela Diaconu

**Affiliations:** European Commission, Joint Research Centre, 2400 Geel, 111 Retieseweg, Belgium; mitja.vahcic@ec.europa.eu (M.V.); Miguel.RUIZ-OSES@ec.europa.eu (M.R.O.); Grzegorz.RARATA@ec.europa.eu (G.R.); Gabriela.DIACONU@ec.europa.eu (G.D.)

**Keywords:** simulants, explosives detection, aviation security, testing, harmonisation, standardisation, X-ray

## Abstract

Explosives detection systems (EDS) based on X-ray are used at airports to screen baggage for the presence of explosives. In Europe and the United States, EDS equipment is tested extensively by specialist test centres prior to approval for operational use in airports. Once EDS are installed in airports, however, it can be challenging to test the EDS equipment and verify that it continues to perform at the highest level, because of the impracticality of introducing bulk explosives into civil aviation airports. We have developed inert, non-toxic polymer-bonded simulants and validated them against real explosives using EDS equipment. The accuracy of our simulants is within 1% of the target bulk density, and within 2% of the target effective atomic number, and the materials have a stability of at least 4 years, with an uncertainty of 0.5%. The simulants generate alarms in almost 100% of cases on a wide range of commercial EDS models, and we consider the simulants fit for purpose for use during testing of EDS equipment at airports.

## 1. Introduction

In the last 40 years there have been many incidents involving explosives onboard aircraft. The three deadliest incidents were Air India Flight 182 [1] with 329 casualties in 1985, Pan Am Flight 103 [2] with 270 casualties in 1988 and Metrojet Flight 9268 [3] with 224 casualties in 2015. In addition to the human cost, there are significant and complex economic consequences of terrorism in aviation [4]. The physiological impact is also considerable; during the period 2014–2019, terrorism was consistently identified by citizens as one of the top five important issues facing the European Union (EU) [5].

In Europe, civil aviation security is regulated at EU level under Framework Regulation 300/2008 [6] and its related supplementing and implementing legislation [7]. The detailed measures for the implementation of these basic standards are laid down in the Commission Implementing Regulation (EU) 2015/1998 [8]. This legislation includes minimum performance requirements for security screening equipment, including explosives detection systems used to screen baggage for the presence of explosives. In aviation security, explosives detection systems (EDS) is a specific term that refers to X-ray equipment with the functionality to automatically detect (and indicate by means of an alarm) the presence of explosives in screened baggage. EU legislation requires that all baggage has to be screened before being loaded onto the aircraft hold. Although multiple screening solutions are permitted by legislation, the sheer number of bags and the limited time available for screening mean that in practice almost all items of hold baggage are screened by EDS. 

State-of-the-art explosives detection systems deployed in airports today are commonly based on dual-energy and/or computer tomography. At the typical energy range of baggage screening equipment (160 keV), the attenuation is determined by two processes, namely Compton scattering and photoelectric absorption. The relative contributions of these processes depend on the energy and atomic properties of scanned items [7], and dual energy systems exploit this phenomenon to provide better discrimination between explosives and benign items based on both density and atomic composition. Discrimination is improved further when combined with volume imaging such as computer tomography (CT). More information about the technological development of X-ray transmission equipment for security screening can be found in [9,10,11]. EDS are deployed as a qualitative, binary method for the rapid primary screening of all hold baggage. As such, it forms the last line of defence against explosives being brought on-board aircraft in hold baggage and is, therefore, an extremely important category of equipment. In Europe and the United States, EDS equipment is tested extensively by specialist test centres [12,13] prior to approval for operational use in airports. 

Once EDS are approved and installed in airports, however, there are limited means to routinely test the EDS equipment and verify that it continues to perform at the highest level, because of the impracticality of introducing bulk explosives into civil aviation airports. The purpose of this work, therefore, is to develop high-quality explosives simulants that can be used as standardised testing materials in European airports to verify the detection performance of EDS. The simulants have to be inert, non-toxic and should mimic the relevant properties of explosives. For transmission X-ray systems, those properties are bulk density and effective atomic number, Z_eff_.

There are a few research groups and companies around the world developing explosives simulants for verifying EDS performance, including (but not limited to) [14,15,16,17,18,19,20]. To have confidence in the conclusions of testing performed during inspections by national or European inspectors, explosives simulants should be supported by impartial and robust data (e.g., composition, accuracy and shelf-life). If such information is not provided together with simulants, most users do not have the technological means to check these parameters themselves. Standardised testing materials also enable stakeholders to compare test results from different equipment across different geographical locations and over time. The outcome of this work should lead—for the first time—to such standardised materials for use in testing across European airports.

The main challenge in this work is that we are aiming at developing standards with material properties as close as possible to explosives, but that one of those properties—effective atomic number—does not itself have a commonly agreed definition. Effective atomic number is a real (non-integer) number describing a hypothetical single element that will give the same X-ray attenuation as the substance being evaluated. There are, however, many different definitions and one paper [21] identifies at least 11 definitions and compares the values obtained from different formulae for a selection of materials. Commercial EDS models implement the concept of effective atomic number in different (and proprietary) ways, and consequently in this work, we also had to adopt a somewhat experimental approach. This paper describes our approach to developing and producing solid, polymer-bonded simulants. Our conclusion is that the materials are fit for purpose to be used by authorities for verifying the detection performance of EDS equipment during airport testing.

## 2. Results and Discussion

### 2.1. Characterisation of Explosives

There is no common standard for how commercial EDS calculate bulk density and effective atomic number (Z_eff_) from the raw data obtained during scanning of an object. Consequently, the internal values for bulk densities and Z_eff_ differ from one manufacturer to another. For this reason, the only way to validate the properties of simulants is compare them to the properties of real explosives, ideally measured on the same equipment. In this work, the properties of over 40 different explosives were measured using the Nuctech XT2080SI Kylin. This equipment has passed ECAC testing for so-called standard EDS-C2 [12]. The measurements of real explosives were performed at the Instituto Nacional de Técnica Aeroespacial (INTA), Spain, and Nederlandse Organisatie voor Toegepast (TNO), Netherlands, under contract to the Joint Research Centre (JRC). The dataset comprises over 900 screenings to determine average values of effective atomic number and mass density for a range of military, commercial and some homemade explosives. The dataset provides us with the reference characteristics of explosives and enables us to design realistic simulants with accurate X-ray attenuation properties.

### 2.2. Produced Simulants

**Figure 1:**
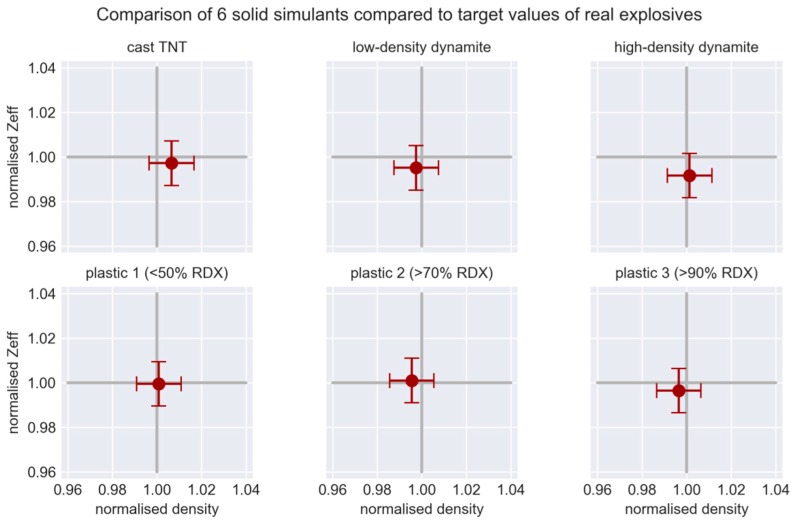
Bulk density and effective atomic number of our simulants normalised to the target values of selected explosives. The error bars are 1% for both density and effective atomic number.

We selected multiple explosives as target points for developing our first simulants. As the intention is for these simulants to be used for field testing of EDS equipment, we do not identify the precise values of density and Z_eff_ here in order to preserve the confidentiality of the testing process. In this paper, we focus on solid simulants mimicking solid explosives. We also developed powder and emulsion simulants, but these involve completely different ingredients and production processes and we intend to describe them in a separate paper. The accuracy (in terms of normalised density and effective atomic number) of six selected solid simulants compared to real explosives is illustrated in Figure 1. 

### 2.3. Stability of Simulants

**Figure 2:**
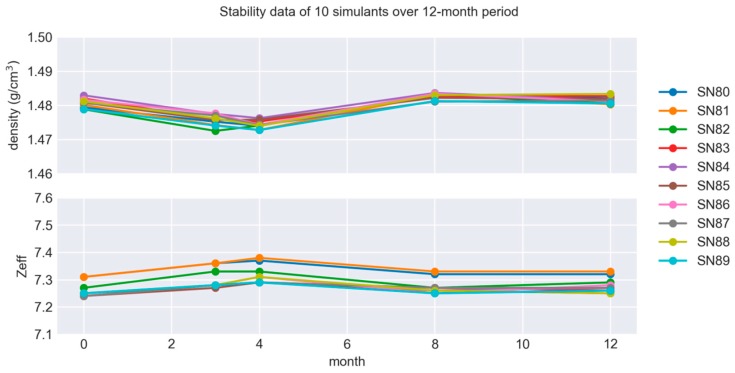
Stability data for 10 produced simulants over a 12-month period. According to the data, the simulants are stable for at least 48 months with an uncertainty of 0.5%. The variability in the above chart is clearly dominated by the equipment fluctuations (see Section 3.5).

Stability testing is necessary to establish conditions for storage as well as conditions for dispatch to the customers. The stability study was carried out using a standard design model where samples are measured at various time points and the linear regression analysis is used at the end to calculate the stability of the samples. The measurements at each time point were performed under repeatability conditions. For determining the stability of the simulants, we tested structural integrity of the simulants along with bulk density and Z_eff_. These parameters were systematically followed for 12 months at room temperature (21 °C). Stability at lower and elevated temperatures were not tested due to well established tolerance of polyurethane (PU) rubbers to heat and cold. Structural stability testing of polymer bonded simulants shows that the simulants have good stability with no visual changes observed during the test time period. Stability calculations indicate stability greater than 48 months for both Z_eff_ and for bulk mass density at an uncertainty of 0.5% (see Figure 2). All together, these results confirm the good long-term stability of the simulants. Stability was tested for a bulk plastic explosive simulant and is expected to be similar for other simulated materials because of similar material composition.

### 2.4. Measurement Uncertainty

To estimate the measurement uncertainty, we followed the Guide to the Expression of Uncertainty in Measurement [22]. We identified four main contributors associated with combined measurement uncertainty: homogeneity, intermediate precision, long-term material stability and long-term instrument stability. Our calculations show that the expanded measurement uncertainty with a coverage factor of k = 2, corresponding to a level of confidence of about 95% is 0.2% for bulk density and 0.3% for Z_eff_. 

Based on subject matter expertise and experience with development of certified reference materials, and considering our calculation does not account for geometry effects, and potential system-to-system variability, and possibly other factors, we decided to adopt a conservative approach and increase the final measurement uncertainty from 0.2% to 1.0% for bulk density and from 0.3% to 2.0% for Z_eff_.

### 2.5. Detection of Simulants Using EDS

**Figure 3:**
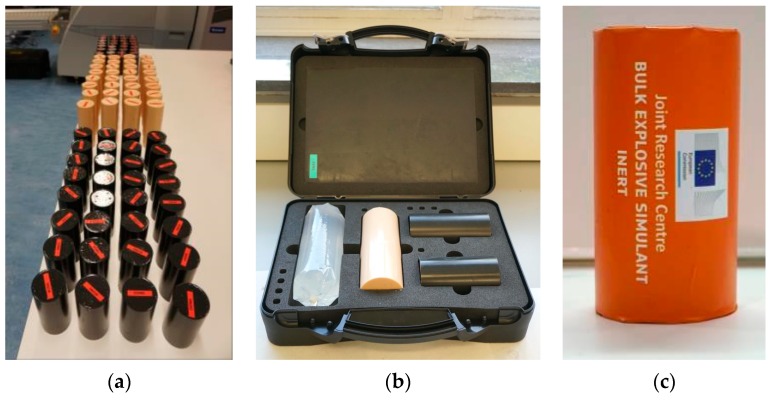
Examples of our explosives simulants showing: (**a**) standardised dimensions of cast materials; (**b**) materials housed in a tailored plastic case for detection testing; (**c**) prototype encapsulation and labelling.

After measuring the density and effective atomic number of six solid simulants, they were placed in a plastic cases (model T4N) with dimensions 341 mm by 252 mm by 100 mm from Rajapack (Belgium). Foam inserts to hold the simulants were custom designed and produced for us by Hoffmann Group (Germany). An example is shown in Figure 3. 

Simulants were scanned by a variety of commercial EDS at the JRC’s laboratory in Geel, Belgium and also at several international European airports in cooperation with the relevant authorities. The detection testing was performed using the following nine EDS models: eXaminer 3DX, eXaminer XLB, eXaminer SX, VIS-HR (L3 Technologies Inc.), HI-SCAN 10080 XCT, HI-SCAN 10080 EDX-2is, HI-SCAN 10080 EDtS (Smiths Detection), Kylin XT2080SI (Nuctech), CT-80DR (Reveal Imaging Technologies). During detection testing, we did not measure material characteristics, only whether the simulant caused an alarm on the EDS or not. The results are shown in Table 1.

### 2.6. Detection of Doped Simulants Using ETD

In addition to the simulants we developed for verifying the detection performance EDS based on transmission X-ray, we also experimented with the development of doped simulants. We wanted to develop dual-purpose simulants that can be used for verifying both EDS detection performance and also explosive trace detection (ETD) performance by swabbing the outer surface of the simulant. 

**Figure 4:**
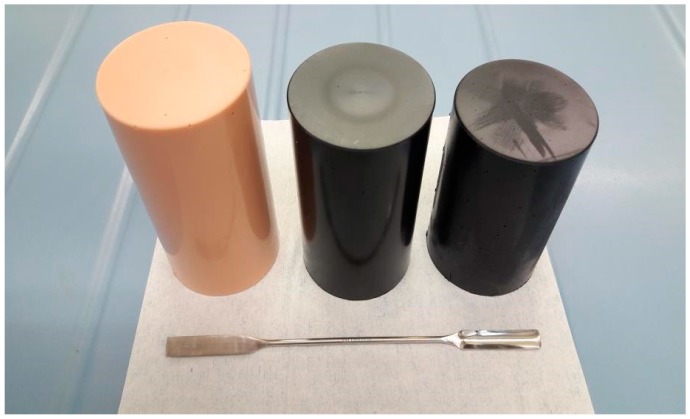
Dual-purpose simulants doped with 1% (*w*/*w*) PETN, RDX, and EGDN (from left to right), for verifying the detection performance of both EDS and explosive trace detection (ETD) equipment.

For this reason, we developed three proof-of-concept simulants, doped with 1% (*w*/*w*) pentaerythritol tetranitrate (PETN), 1,3,5-trinitro-1,3,5-triazinane (RDX) and ethylene glycol dinitrate (EGDN) explosives (see Figure 4). These three explosives cover three main families of explosives used for military and industrial applications. The simulants were tested with five commercial ETD systems and the results are shown in Table 2. 

Detection with ETD equipment was good (sometimes 100%) for the PETN- and RDX-doped simulants, but less satisfactory for the EGDN-doped simulant. EGDN is normally detected 100% of the time with ETD, but since EGDN is an immiscible liquid it did not mix well with the polyurethane matrix, resulting in seams of EGDN distributed too sparsely throughout the simulant. We conclude that PETN and RDX are more suitable for producing doped simulants based on polyurethane binders.

## 3. Materials and Methods

### 3.1. Software Modelling

Effective atomic number is a critical concept for the automated detection of explosives using dual-energy, X-ray equipment. Along with density, these two parameters are the primary features used by software algorithms to detect explosives in scanned baggage. However, effective atomic number is a (semi-) empirical parameter, and there are multiple definitions in literature [21]. Manufacturers of commercial explosive detection systems implement proprietary solutions—each with its own characteristics that depend on hardware (X-ray sources, detectors, etc.) and software. 

In this work, we adopted a definition and algorithm for determining effective atomic number developed by the Lawrence Livermore National Laboratory [23,24]. This definition, referred to as Z_e_ (to differentiate from Z_eff_) has several advantages, namely it is based on a physical model instead of an empirical one, it comprises one fixed algorithm over a range of materials and energy spectra of interest, and that each value of the parameter corresponds to a well-defined x-ray absorption behaviour. We developed our own implementation [25] using Microsoft Excel 2010 to calculate the LLNL definition of effective atomic number. We used this software to screen substances for their potential use as simulants ingredients, and also to (partially) calibrate the system-dependent parameter values returned by the commercial EDS. 

### 3.2. Materials

All simulants described in this paper were cylindrical with a diameter of 60 ± 1 mm. The length of simulants depended on their composition and mass. The main components used for the construction of simulants were boron carbide powder (B_4_C) (Alfa Aesar, Kandel, Germany), aluminium oxide powder (Al_2_O_3_) (VWR, Leuven, Belgium), lithium carbonate powder (Li_2_CO_3_) (VWR, Leuven, Belgium) and liquid polyurethane rubber, VytaFlex^®^ 30 series (Smooth-On, Macungie, PA, USA), with a shore hardness of 30A according to ASTM D-2240. For the production of negative silicone moulds, OOMOO 30 (Smooth-On, Macungie, USA) liquid silicone rubber with shore hardness of 30A was used.

For long-term stability studies and calibration purposes, pure polyoxymethylene (Delrin), polytetrafluoroethylene (PTFE), graphite, silicon and aluminium rods with the diameter of 60 mm and length of 200 mm, from American Elements (Los Angeles, CA, USA) were used.

Chemicals used for synthesis of explosives for doping of simulants were: concentrated nitric and sulphuric acids, hexamethylenetetramine and ethylene glycol (all Merck, Darmstadt, Germany), pentaerythritol (Alfa Aesar, Kandel, Germany) and deionized water.

### 3.3. Production Equipment

The Turbula^®^ benchtop 3D mixer (WAB, Muttenz, Switzerland) was used to homogenize filler powder components (B_4_C, Al_2_O_3_, Li_2_CO_3_). A screwcap polypropylene container was used to minimize the contamination of the final mixture with elements heavier than carbon due to abrasive properties of some powders used. A vacuum degassing chamber was used to degas the liquid rubber simulant mixture before casting. Plexiglas moulds and shape positives developed and manufactured in-house were used to produce silicon moulds which were in turn used to produce the final cylindrical simulants mirroring the shape of the Plexiglas positives.

### 3.4. Production Process

The process of bulk explosive simulant production required combining of liquid polyurethane (PU) rubber binder with premixed and homogenized powder filler components. The liquid simulant mixtures were homogenized by using high powered mixer for 10 min and subsequently degassed at −1 bar before they were cast into a specific shape using pre-prepared silicon moulds. The liquid rubber simulant mixtures were then left to cure in a dry atmosphere for at least 16 h. After this time period the cured simulants were demoulded and measured using dual-energy, computer-tomography equipment. Explosives for doping of simulants were synthesised in-house at JRC’s laboratories according to established literature procedures.

### 3.5. Measurement Equipment

The density and effective atomic number of the target energetic materials and our simulants were determined using commercial EDS, namely XT2080SI Kylin (Nuctech Company Limited, Beijing, China) and HI-SCAN 10,080 XCT (Smiths Detection, London, UK). Both systems have passed testing by the European Civil Aviation Conference [12] and are deployed operationally in airports. Such EDS equipment is designed to indicate the presence/absence of explosives inside scanned baggage. It is not intended, however, to be used for quantitative measurements of material properties. Furthermore, the data processing and detection algorithms are trade secrets. For both EDS equipment used in this work, the manufacturers kindly made available software tools to extract values of density and effective number for scanned materials.

**Figure 5:**
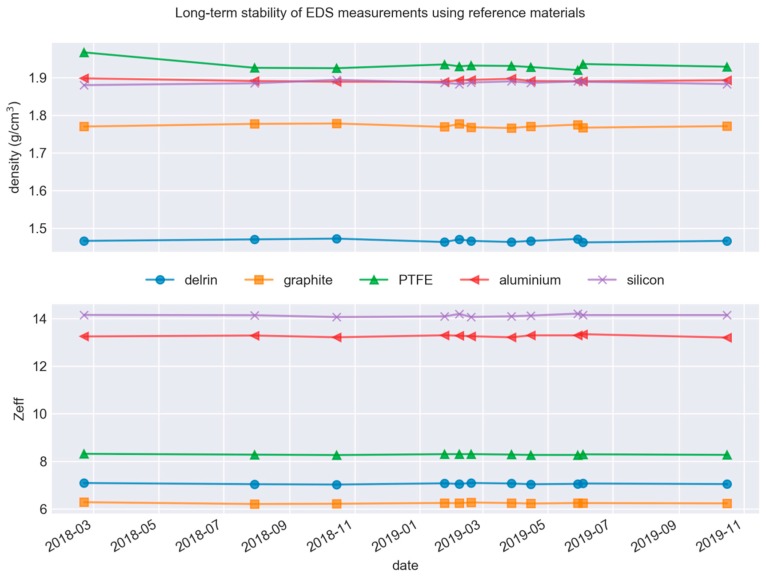
Long-term stability of equipment.

The long-term signal stability of the EDS was determined by measuring five calibration pieces every few months. The results show the uncertainty of 0.3%–0.5% for Z_eff_ and 0.2%–0.3% for mass density, with the instruments fluctuating slightly around a central value but otherwise stable. The stability data is shown in Figure 5.

For ETD swabbing of doped simulants (see Section 2.6), a QS-B220 instrument (L3, New York City, NY, USA), ITEMISER 4DX instrument (Rapiscan, Salfords, UK), ITEMISER DX (Rapiscan, previously Morpho Detection, Salfords, UK), IONSCAN 500DT (Smiths Detection, London, UK) and IONSCAN 600 (Smiths Detection, London, UK), were used. The configurations (hardware and algorithm version) of the ETD equipment were those that passed ECAC testing [12].

### 3.6. Calibration of Measurement Equipment

#### 3.6.1. Reference Materials Approach

**Figure 6:**
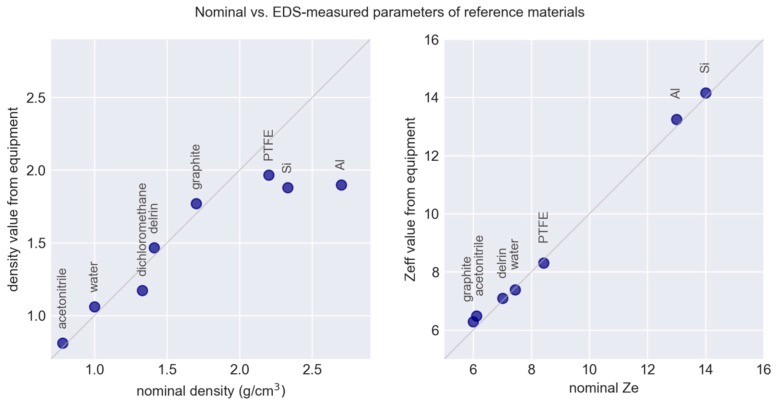
Nominal vs. measured values of density (**left**) and effective atomic number (**right**) for selected reference materials measured on a commercial EDS.

Our initial idea was that recipes for simulants would be developed using software modelling of density and Ze, and a calibration curve between theoretical Ze values and instrument-specific Z_eff_ values using physical calibrants would be used to correct or fine-tune the final properties of the simulant. We attempted to calibrate the values obtained by the EDS equipment by scanning standardized pieces of pure materials with known densities and theoretical Ze numbers. We noticed that items with the same composition, but different geometry could have different values, so decided to standardise the calibrants (and our subsequent solid simulants) as cylinders with a diameter of 60 mm. We procured five calibration pieces of high purity made of Delrin, PTFE, graphite, aluminium and silicon, and the resulting calibration chart in shown in Figure 6.

As can be seen from Figure 6, there were, however, a number of limitations with this approach. Firstly, experience showed that a simple linear calibration between nominal Ze and the Z_eff_ values measured by the equipment is overly simplistic. The calibration depends on density, shape, and possibly other parameters. Secondly, finding suitable calibrants was difficult, as readily available, high-purity materials are not necessarily in the correct range for explosives or in the appropriate physical form. Thirdly, the chemical composition of our selected polyurethane binder was not known exactly, hence the theoretical value for modelling could not be calculated precisely. For this reason, we adopted an experimental design approach to calibration, as described in the next section.

#### 3.6.2. Experimental Design Approach

To develop a calibration using representative materials that takes account of the interaction between density and effective atomic number, we produced 9 simulants that, to the best of our prior knowledge, would be evenly spaced out in the 2D-parameter space of density and effective atomic number. Each simulant had a precisely known composition of three ingredients (e.g., binder, B_4_C, Al_2_O_3_), given in Table 3.

**Figure 7:**
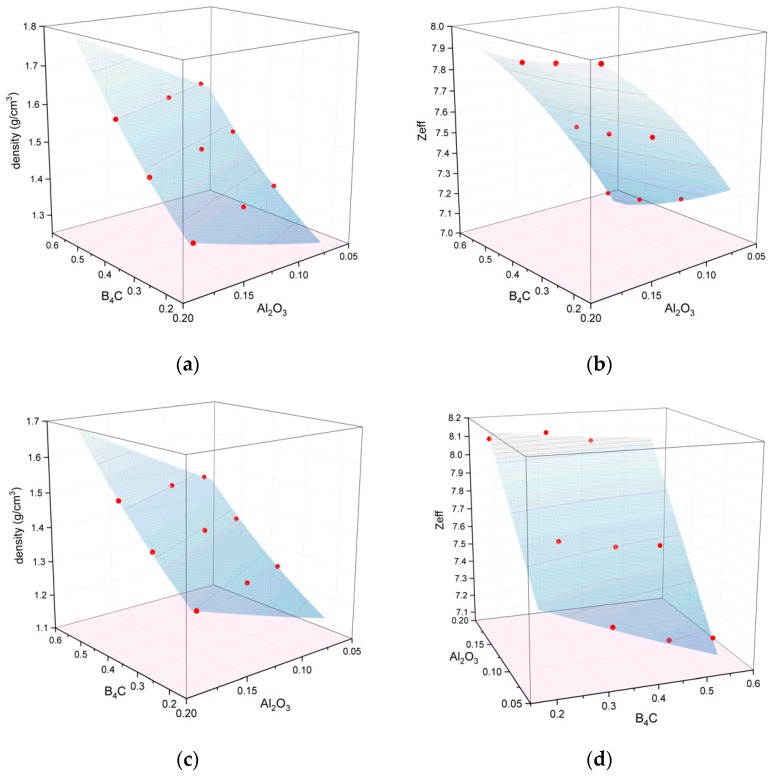
Fitted surfaces (blue) derived from a matrix of 9 simulants (red dots), used as an empirical calibration between simulant composition and instrument-specific measurements of material characteristics: (**a**) density measured on the Nuctech Kylin; (**b**) Z_eff_ measured on the Nuctech Kylin; (**c**) density measured on the Smiths XCT; (**d**) Z_eff_ measured on the Smiths XCT.

The simulants were measured in the EDS equipment and experimental values of density and effective atomic number obtained. The data were then plotted as points in “ingredient space”, i.e., as a 3D surface where the x and y axes represent the proportions of two of the three ingredients, and the *z*-axis the measured parameters (density or Z_eff_). The proportion of the third ingredient is not an independent variable, as it is simply the difference between 100% and the sum of the other two proportions. Using the Origin 2018 software (OriginLab Corporation, Northampton, MA, USA), equations for the 3D surfaces were determined. A 2D dimensional polynomial was chosen for the surface, in order to account for interactions whilst minimising the potential for overfitting. The calibration surfaces are shown in Figure 7 and the fitting parameters are given in Table 4. These 3D surfaces provided an empirical calibration between simulant composition and EDS measurement data, and they turned out to be a time-saving and effective means to determine the correct proportions of ingredients to match the characteristics of explosives measured previously on the same equipment.

### 3.7. Repeatability and Intermediate Precision

Measurement repeatability for the bulk mass density and Z_eff_ was evaluated by measuring a single simulant of a plastic explosive consecutively 10 times in a single day and calculating the relative standard deviations. Measurement repeatability was RSD = 0.14% for density and RSD = 0.19% for Z_eff_.

**Figure 8:**
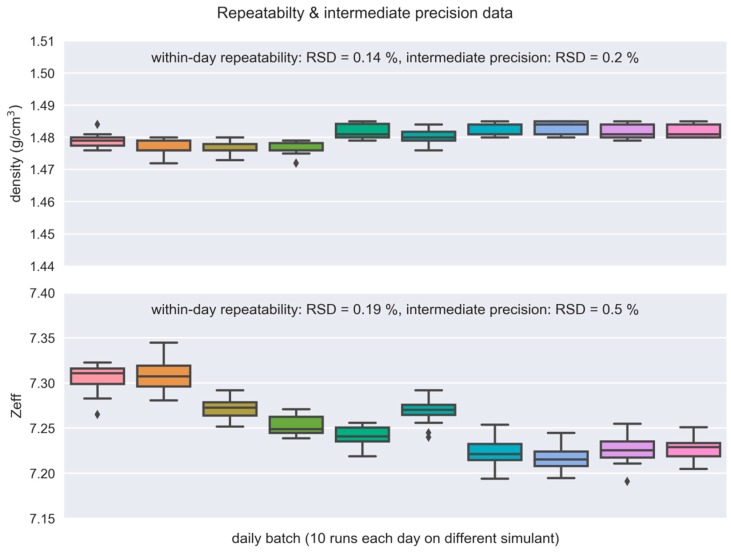
Measurements of 10 different simulants measured 10 times on different days to calculate intermediate precision of density (**top**) and effective atomic number, Z_eff_ (**bottom**). The intermediate precision incorporates repeatability, day-to-day equipment fluctuations, and the variability in the simulant production process. (The box extends from first to third quartiles (Q1, Q3) of the data. The position of the whiskers is 1.5 * (Q3-Q1) from the edges of the box. Outlier points are those past the end of the whiskers.)

Intermediate precision was evaluated on ten independently produced replicates of bulk plastic-explosives simulants with the same compositions, each measured 10 times on different days. Expressed as relative standard deviation (RSD), intermediate precision for bulk mass density was 0.2% and 0.5% for Z_eff_. The data are shown in Figure 8.

## 4. Conclusions

We acquired characterisation data of different categories of explosives and produced solid simulants by combining a liquid polyurethane rubber binder with premixed and homogenized powder filler components. The simulants were validated by comparing their characteristics directly with those of bulk explosives using the same equipment. We determined the accuracy of our simulants to be within 1% of the target density, and within 2% of the target effective atomic number, and the materials are considered stable for at least 4 years. The simulants generate alarms almost 100% of the time when screened by a selection of commercially available EDS currently deployed in European airports. In conclusion, the simulants are deemed fit for purpose for aviation security inspectors to verify that EDS equipment is functioning satisfactorily in terms of its detection performance. We also developed proof-of-concept simulants that were doped with 1% w/w of explosives and are suitable as dual-use materials for verifying both EDS and ETD equipment at security checkpoints.

## Figures and Tables

**Table 1:** Detection results (expressed as alarms/trials) for six simulants when passed through nine different commercial explosive detection systems (EDS).

Equipment ^1^	SN59	SN60	SN62	SN64	SN67	SN69
**EDS 1**	20/20	20/20	20/20	20/20	20/20	20/20
**EDS 2**	20/25	23/25	16/25	20/20	20/20	20/20
**EDS 3**	20/20	20/20	12/20	16/20	20/20	20/20
**EDS 4**	20/20	20/20	20/20	10/10	10/10	10/10
**EDS 5**	20/20	19/20	19/20	20/20	20/20	20/20
**EDS 6**	10/10	10/10	8/10	10/10	10/10	10/10
**EDS 7**	20/20	20/20	20/20	20/20	20/20	20/20
**EDS 8**	20/20	20/20	3/20	20/20	17/20	20/20
**EDS 9**	10/10	10/10	10/10	10/10	10/10	10/10

^1^ The explosives detection system (EDS) equipment is listed in the body text, but the results are provided anonymously to preserve the confidentiality of the equipment performance.

**Table 2:** ETD detection results when swabbing our doped simulants.

Doped Simulant	ETD 1 ^1^	ETD 2	ETD 3	ETD 4	ETD 5
PETN in ‘PETN’ simulant	6/6	6/6	4/6	6/6	3/6
RDX in ‘SEMTEX-H’ simulant	6/6	6/6	6/6	6/6	6/6
EGDN in ‘commercial dynamite’ simulant	4/6	0/6	0/6	0/6	2/6

^1^ The ETD instruments are identified in Section 3.5, but the results are provided anonymously.

**Table 3:** Composition of the nine simulants used to generate calibration surfaces.

Simulant	Binder	B_4_C	Al_2_O_3_
SN-43	48.0%	44.8%	7.2%
SN-44	37.7%	54.7%	7.5%
SN-45	60.7%	32.6%	6.7%
SN-46	50.9%	30.5%	18.5%
SN-47	63.5%	18.2%	18.3%
SN-48	40.6%	40.7%	18.7%
SN-50	38.9%	48.9%	12.1%
SN-51	61.8%	26.6%	11.6%
SN-52	49.2%	38.9%	11.9%

**Table 4:** Fitting parameters for our calibration surfaces obtained using Origin 2018 software.

	Nuctech Kylin		Smiths XCT	
	Density	Z_eff_	Density	Z_eff_
Equation	z = z_0_ + a·x + b·y + c·x^2^ + d·y^2^ + f·x·y			
Z_0_	1.14036	6.92839	1.02539	7.35118
a	0.24857	−1.7336	0.29455	−1.57553
b	0.2946	9.77275	0.63816	4.4915
c	0.73168	0.81162	0.59796	0.25238
d	1.24272	−19.0843	0.75723	0.22944
f	1.82343	5.00551	1.68086	6.25114
Adj. R-Square	0.9999	0.99847	0.99996	0.99499
